# Supplementary material for: Hybrid Models and Biological Model Reduction with PyDSTool
Source: PLoS Comput Biol. 2012 Aug 9;8(8):e1002628. doi: 10.1371/journal.pcbi.1002628 (PMC3415397; doi:10.1371/journal.pcbi.1002628)
Supplement: Text S4 — Complete source code for the PyDSTool package (version 0.88.120504). Includes API documentation and help files linking to web pages. This file is identical to the current public release on Sourceforge.net. (ZIP) [file pcbi.1002628.s004.zip › PyDSTool/html/PyDSTool.Toolbox.optimizers.line_search.fibonacci_section-pysrc.html]

xml version="1.0" encoding="ascii"?


PyDSTool.Toolbox.optimizers.line\_search.fibonacci\_section


| Home | Trees | Indices | Help | PyDSTool |
| --- | --- | --- | --- | --- |

Package PyDSTool ::
Package Toolbox ::
Package optimizers ::
Package line\_search ::
Module fibonacci\_section

[hide private]

[frames] | no frames]

# Source Code for Module PyDSTool.Toolbox.optimizers.line\_search.fibonacci\_section

```
 1   
 2  # Matthieu Brucher 
 3  # Last Change : 2007-08-26 19:48 
 4   
 5  """ 
 6  Line Search with the Fibonacci section method 
 7  """ 
 8   
 9  import math 
10   


11 -class FibonacciSectionSearch(object):


12    """ 
13    Line Search with the Fibonacci section method, optimal section method 
14    """ 


15 -  def __init__(self, min_alpha_step, alpha_step = 1., **kwargs):


16      """ 
17      Needs to have : 
18        - a minimum step size (min_alpha_step) 
19      Can have : 
20        - a step modifier, a factor to modulate the step (alpha_step = 1.) 
21      """ 
22      self.minStepSize = min_alpha_step 
23      self.stepSize = alpha_step 
24   
25      limit = self.stepSize / self.minStepSize 
26      self.fibonacci = self.computeFibonacci(limit)

27   


28 -  def __call__(self, origin, function, state, **kwargs):


29      """ 
30      Returns a good candidate 
31      Parameters : 
32        - origin is the origin of the search 
33        - function is the function to minimize 
34        - state is the state of the optimizer 
35      """ 
36      direction = state['direction'] 
37      ak = 0 
38      v_ak = function(origin + ak * direction) 
39      if 'initial_alpha_step' in state: 
40        bk = state['initial_alpha_step'] 
41      else: 
42        bk = self.stepSize 
43      v_bk = function(origin + bk * direction) 
44   
45      k = 1 
46   
47      uk = ak + (self.fibonacci[-2] / self.fibonacci[-1]) * (bk - ak) 
48      v_uk = function(origin + uk * direction) 
49      lk = ak + self.fibonacci[-3] / self.fibonacci[-1] * (bk - ak) 
50      v_lk = function(origin + lk * direction) 
51   
52      while True: 
53        if v_uk < v_lk: 
54          if k > len(self.fibonacci) - 2: 
55            state['alpha_step'] = uk 
56            return origin + uk * direction 
57          ak = lk 
58          v_ak = v_lk 
59          lk = uk 
60          v_lk = v_uk 
61          uk = ak + self.fibonacci[-k - 1] / self.fibonacci[-k] * (bk - ak) 
62          v_uk = function(origin + uk * direction) 
63        else: 
64          if k > len(self.fibonacci) - 2: 
65            state['alpha_step'] = lk 
66            return origin + lk * direction 
67          bk = uk 
68          v_bk = v_uk 
69          uk = lk 
70          v_uk = v_lk 
71          lk = ak + self.fibonacci[-k - 2] / self.fibonacci[-k] * (bk - ak) 
72          v_lk = function(origin + lk * direction) 
73        k = k + 1

74   


75 -  def computeFibonacci(self, limit):


76      fibonacci = [1., 1.] 
77      while (fibonacci[-1] < limit): 
78        fibonacci.append(fibonacci[-2] + fibonacci[-1]) 
79   
80      return fibonacci

81
```

  


| Home | Trees | Indices | Help | PyDSTool |
| --- | --- | --- | --- | --- |

|  |  |
| --- | --- |
| Generated by Epydoc 3.0.1 on Fri May 4 15:24:25 2012 | http://epydoc.sourceforge.net |
